# Atrial Fibrosis by cardiac MRI is a correlate for atrial stiffness in patients with atrial fibrillation

**DOI:** 10.21203/rs.3.rs-2818190/v1

**Published:** 2023-04-18

**Authors:** Jérôme Lamy, Roy Taoutel, Romy Chamoun, Joseph Akar, Steven Niederer, Hamid Mojibian, Steffen Huber, Lauren A. Baldassarre, Judith Meadows, Dana C. Peters

**Affiliations:** Yale University; Yale University; Yale University; Yale University; Kings College London; Yale University; Yale University; Yale University; Yale University; Yale University

**Keywords:** magnetic resonance imaging, strain, atrial fibrillation, atrial fibrosis, pressure, late gadolinium enhancement, left atrium

## Abstract

**Aims:**

A relationship between left atrial strain and pressure has been demonstrated in many studies, but not in an atrial fibrillation (AF) cohort. In this work, we hypothesized that elevated left atrial (LA) tissue fibrosis might mediate and confound the LA strain vs. pressure relationship, resulting instead in a relationship between LA fibrosis and stiffness index (mean pressure/LA reservoir strain).

**Methods and Results:**

Sixty-seven patients with AF underwent a standard cardiac MR exam including long-axis cine views (2 and 4-ch) and a free-breathing high resolution three-dimensional late gadolinium enhancement (LGE) of the atrium (N=41), within 30 days prior to AF ablation, at which procedure invasive mean left atrial pressure (LAP) was measured. LV and LA Volumes, EF, and comprehensive analysis of LA strains (strain and strain rates and strain timings during the atrial reservoir, conduit and active phases) were measured and LA fibrosis content (LGE (ml)) was assessed from 3D LGE volumes. LA LGE was well correlated to atrial stiffness index (LA mean pressure/LA reservoir strain) overall (R=0.59, p<0.001), and among patient subgroups. Pressure was only correlated to maximal LA volume (R=0.32) and the time to peak reservoir strain rate (R=0.32), among all functional measurements. LA reservoir strain was strongly correlated with LAEF (R=0.95, p<0.001) and LA minimum volume (r=0.82, p<0.001).

**Conclusion:**

In our AF cohort, pressure is correlated to maximum LA volume and time to peak reservoir strain. LA LGE is a strong marker of stiffness.

## Introduction

Atrial fibrillation (AF) is the most common arrhythmic disease at 1 to 2% of the U.S population, with the forecast of increasing prevalence [17]. AF originates from a trigger and substrate, with substrate being a large fibrotic atrium. As atrial fibrosis stiffens the LA [5], this may cause additional increased atrial pressure, or reduced LA strain. The role of elevated cardiac pressure in AF and left atrial (LA) remodeling is important [32, 39]. Firstly, elevated pressure is thought to increase LA volumes, ultimately leading to stretch and to development of atrial fibrosis [33], and potentially lead to new onset AF [22]. Secondly, many categories of patients with elevated cardiac pressure progress to AF, including patients with heart-failure [1], hypertrophic cardiomyopathy [26], and obstructive sleep apnea [21] among others.

The triad of strain, pressure and stiffness (e.g. fibrosis) are crucial in understanding atrial remodeling. A simple to understand model of strain (e, equals stress / stiffness) on a spherical shell of radius r, and thickness b, yields [34, 37]:

Eq. 1. $$\in \propto \frac{P•r}{\left(b•E\right)}$$

This equation describes a relationship between strain (e), pressure (P), stiffness (E, atrial fibrosis, etc.) and volume (r^3^). The equation also explains the origin of left atrial “stiffness index” (E∝ P/*ϵ*_*LA*_) (pressure/LA reservoir strain) [4] [16] employed by echocardiography (using E/e’ for pressure). Atrial stiffness is partly attributable to atrial fibrosis, which can be evaluated using 3D high resolution late gadolinium enhancement (LGE) [29] with cardiac magnetic resonance (CMR). Increased atrial LGE (fibrosis causing increased stiffness) is associated with reduced LA function (both strain and EF), as confirmed by many studies [6, 11, 12, 13, 19, 27, 30].

In non-AF subjects, atrial strain changes are often advocated as an early sign of pressure increase [3, 4, 14, 35, 36]. LA strain as a correlate of pressure is an attractive possibility. To date, there are no imaging biomarkers to estimate cardiac pressure accurately; many echo correlates (E/e’, etc) are not fully robust. Others have studied LA strain as a direct biomarker of LV filling pressure [3, 4], or its role in grading diastolic dysfunction (itself a disease indicative of elevated pressure) [35]. The pressure-strain relationship varied from strong to modest [3] [4] [14] [36]. These findings have not been attempted or replicated in an AF cohort. We hypothesized that pressure-strain relationships may be modulated in AF cohort, because of the range of LA volumes and atrial fibrosis, as is suggested by Eq. 1. We examined the relationship of LA strain with elevated pressure in an AF cohort, with the hypothesis that *atrial stiffness* might impact this relationship.

Even while LA strain is an area of increasing interest, it is rarely noted the very tight correlations between LA strain, LA volume, and LA EF. In AF, where LA volumes span a wide range, this becomes even more important. Complete strain analysis (including strains in three phases, systolic, early diastolic and during the atrial kick; also strain rates, and timings of peak strains and strain rates) is not often performed and the unique contributions of these metrics are not known.

## Materials And Methods

### Population

This study is a single center retrospective study and recruited 107 consecutives AF patients (between 2013 and 2020) having a CMR exam including LGE acquisition with no prior ablation scheduled for pulmonary vein ablation procedure within 1 month, at which time average left atrial pressure was directly measured. The study was approved by Yale institutional review board, as exempt chart review study; informed consent was not required. Exclusion criteria were uninterpretable CMR (n = 33, including 23 due to AF during the acquisition, 10 with foreshortened LA views), and more than mild mitral regurgitation (n = 7). A total of 67 patients were included among whom 41 one had a 3D LGE acquisition covering the LA, available and interpretable.

### Cardiovascular Magnetic Resonance Imaging (CMR)

Patients underwent a standard CMR exams, on a 1.5T Siemens magnet (one subject at 3T). Each subject had a conventional CMR including: balanced steady-state free precession (bSSFP) acquisition 4- and 2-chamber views as well as a standard short-axis stack and a 3D LGE exam covering the LA and the LV. bSSFP data were acquired during breath-hold, with the following scan parameters: acquisition matrix 175×210, echo time (TE) 1.4ms, repetition time (TR) 2.8ms, flip angle 50°, pixel size 1.9×1.9mm^2^, slice thickness 8mm. LGE acquisition parameters were [28]: 3D gradient echo inversion recovery (1RR between inversions) with fat-saturation and navigator gating (trailing). ECG-gating with acquisition in ventricular diastole, ~ 150 to 200ms, TR/TE/q = 5ms/1.8ms/15, field of view 340 × 340 mm^2^, slice-thickness 3mm reconstructed to 1.5mm, 0.7 × 0.7mm^2^ in-plane resolution after zero-filling, 592 Hz/pixel, Grappa factor 2, ~ 5 minutes total acquisition time. The inversion time was set by a customized TI-scout to null normal LV myocardium.

### Comprehensive Volumes and Strains

**Figure 1:**
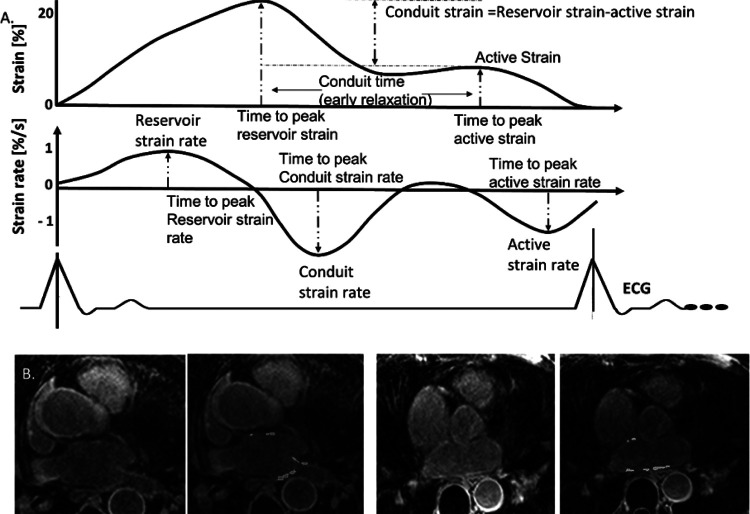
Left atrial strain and fibrosis measurement. A) Comprehensive left atrial strain measurements evaluated nine metrics of strain. These consisted of strain at three phases (reservoir, conduit and, active phase), and timings to peak strain. Additionally, the time derivative of the strain curve yielded three strain rates and times to peak strain rate in the three phases. Timings were normalized by RR. B) In two subjects, atrial LGE slices from a 3D volume are shown with segmentations (in green), using a subject specific threshold, determined by including signal associated with the valvular enhancement, but excluding blood pool signals.

A dedicated previously validated feature tracking software (Cardiotrack [8, 20]) was used to analyze LA longitudinal strain values from 2- and 4-chambers long axis cine views, for this research study. An expert with greater than 7 years of experience in atrial strain evaluation measured three phasic LA strains, and three strain rates in the reservoir (passive), conduit (early diastole) phase, and active contraction (booster) phase of the LA, and the time indices of these phases (both time of peak strain and strain rate for each phase, all normalized by RR) (Fig. 1A). The conduit strain is measured by the difference of those two peaks. The conduit strain time is the duration of time measured between these two peaks.

In all subjects, when possible, three LA volumes (LAVi) (minimum, maximum and pre-atrial kick volumes) were measured using the bi-plane area method [40], and two EFs (active and total). Active LAEF was defined here as (LAV_act_-LAV_min_)/LAV_min_. LAV_act_ is the volume at begin atrial-systole. LA stroke volume (LASVi) was considered as the difference between the maximum and minimum LA volumes. Standard LV volumetric parameters of EF, mass, end-diastolic volume (EDVi), end-systolic volume (ESVi), stroke volume (SVi) were measured. Volumes and mass were indexed by patients’ body surface area (BSA). The total number of functional correlates analyzed was 25. Additional File 1 shows the analysis of LA strains in one patient. All data (LA LGE, LA strain) was analyzed blinded to other metrics and was performed for research purposes.

### LGE post-processing

Quantitative analysis of the LA LGE was performed by manual segmentation using 3D Slicer, used to indicate the pixels containing LA myocardium on each slice of the 3D volume (Fig. 1B). Pulmonary vein ostia were included, up to 5mm into each PV ostia; enhanced valvular pixels were excluded. Using the manual wall segmentation, the enhanced pixels were determined using a patient-specific threshold. The patient-specific threshold was determined using an image intensity ratio (vs. blood signal) of about 1.4 of mean blood pool signal, as previously described [30]. The threshold was adjusted to a level matching the signal intensity of the enhanced mitral valve (which is known to be partly composed of collagen), but also to exclude blood pool pixels. The inter and intra–observer variability of this approach has been reported [30] (intra-observer variability: bias ± 2SDs = − 0.3% ± 2.9%, ICC = 0.94; inter-observer variability: bias ± 2SDs = 0.6% ± 7.3%, ICC = 0.71). All LGE analysis were performed by a single expert (DCP), with more than 10 years of experience in LA LGE analysis. The volume of segmented enhanced LA myocardium was then calculated (LGE in mls) and also normalized by the total LA myocardium volume (LGE%) [30].

### Pressure measurement.

Invasive left atrial pressure was measured prior to the catheter ablation, during which patients were under general anesthesia. Average atrial pressure was measured from the LA using an 8.5 F sheath following transseptal puncture. The electrophysiologists were blinded to pre-procedural LA LGE findings.

### Stiffness Index

We calculated atrial stiffness index as used in echocardiography, i.e. LA pressure/LA reservoir strain [4] [16], using invasive mean atrial pressure.

### Patient categorizations

Motivated by Eq. 1, and prior studies [14], patients were further characterized by presence or absence of enlarged minimum LAVi (> 30 ml/m^2^) (representing mean LAVI + 3SDs [28]) to further examine the relationships in more homogeneous cohorts. We also investigated categorization by elevated or normal pressure, using the median value from our cohort (LAP = 12 mmHg).

### Statistical analysis

JMP (JMP Pro 15.2, SAS) was used for statistical analysis. Descriptive statistics were expressed as mean ± standard deviation. Any p-value < 0.05 was considered as significant. Comparison between groups was performed with a Mann-Whitney test for continuous variable. Spearman rho was calculated, with correlations of 0.2–0.39 considered weak, 0.40–0.59 as moderate, 0.6–0.79 as strong and 0.8–1 as very strong correlations. This was a hypothesis generating study.

## Results

### Population description

**Figure 2:**
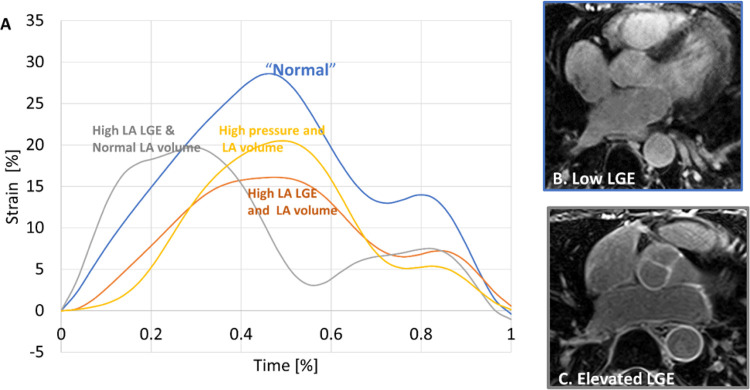
Representative examples of strain and atrial LGE. A) Strain curves for four patients, with normal LA volume, pressure and strain (blue), elevated atrial LGE but normal volume (grey), elevated atrial LGE and high volume (orange), and a patient with high pressure and volume but not significant LGE (yellow). The higher LA volume patients have reduced strains. In the high LA LGE subject with normal volume, strain is also reduced. In the high pressure subject, the time to peak reservoir strain rate increased. B-C) show representative color-coded slices from the atrial LGE images for the normal patient (B), and the patient with elevated LA LGE (C).

The average age of the AF cohort (n = 67) was 61 ± 8 years old, with 45 male patients (67%). Their BMI was 32 ± 6 kg/m^2^ and their BSA was 2.17 ± 0.27 m^2^. Pressure was not highly elevated in our group (12.2 ± 5.2 mmHg). In 23 patients, active function was not observed (either fused E and A waves, or no A wave). LVEF was < 50% in 22% of the subjects (n = 14). Of these, only 4/14 had observed active function. Many (35 of 67) patients had elevated LA volumes (minimum LAVi > 30 ml/m^2^). Table 1 shows evaluated metrics, categorized by minimum LAVi. Figure 2 shows four strain curves and LGE images from the patients. Strain values agree with mean values in other studies of patient groups [18], including AF [11].

### Overview of relationships

**Figure 3:**
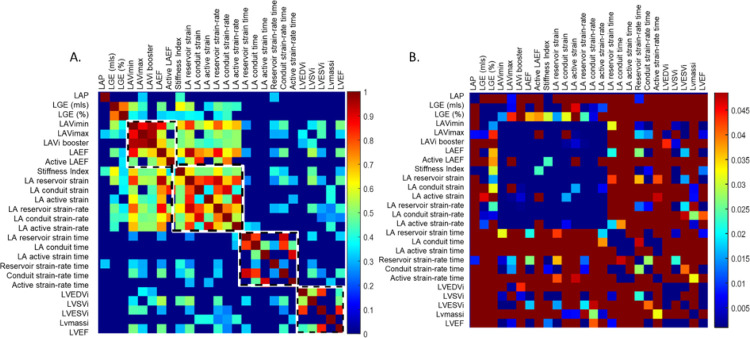
Heat maps, showing significant (p<0.05) Spearman rho correlation coefficients and p-values for 25 parameters within the entire cohort. Non-significant correlations are replaced with R=0. A) Spearman rho correlations. The four squares (with black and white lines) group parameters by LA volumetry; LA stiffness, strain and strain rates; LA deformation timings and LV volumetric parameters. Note that all strain metrics were measured for N=67, except active strain metrics (N=44). Atrial LGE was measured in N=41, and atrial LGE could be correlated with active function metrics in N=36. B) P-value heat map.

Figure 3 shows a heat map of all correlations for the total cohort, with any non-significant correlations plotted as R = 0. Some general trends can be observed. Pressure is not correlated to any functional metrics, with exceptions of maximum LA volumes, and time to peak strain rate in the reservoir phase. Atrial LGE is mostly correlated with LA volumes, strains and most strongly, stiffness index.

Most LA strain and LA volume metrics are strongly correlated (R > 0.6) with each other, except for timings. For example, LAEF correlates with LA reservoir strain, with r = 0.95, p < 0.001. Conventional LV function parameters do not correlate with LA functional parameters strongly.

### Correlations with atrial LGE

**Figure 4:**
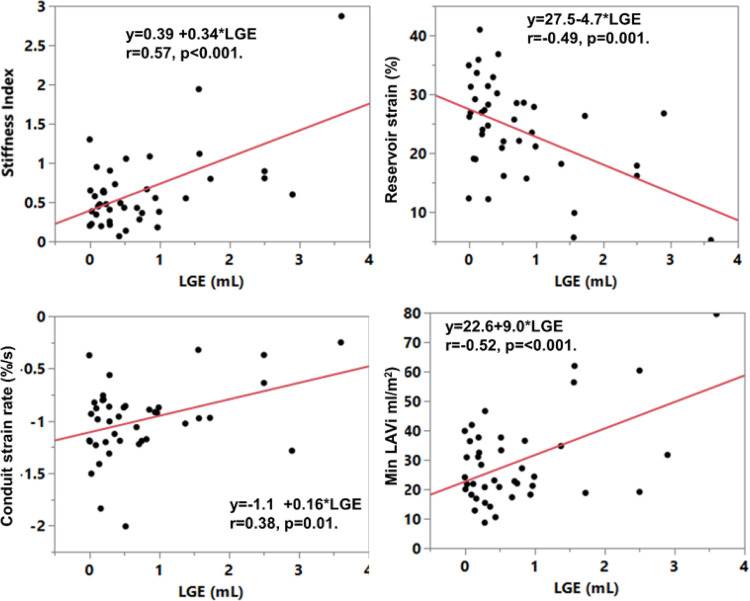
Left atrial fibrosis (LGE (mls)) correlates. LGE was correlated with strains, volumes and strain rates. Correlations were stronger among subjects with normal minimum LA volume. Correlations with Stiffness index was strongest. All shown correlations were significant (p<0.01).

Atrial fibrosis (LGE in mls) was highly correlated to LGE % (R = 0.78) (p < 0.001). Neither LGE (mls) or LGE% correlated with age, but LGE(%) did correlate with BMI (R = 0.54, p < 0.001). Table 2 shows the correlations found with LGE. Atrial fibrosis (LGE mls) correlated most strongly with stiffness index (R = 0.57, p < 0.0001) (Fig. 4, Table 2). When analyzing patients with normal and elevated minimum LAVi separately (Table 2), we found that LA LGE more strongly correlated with metrics of strain in patients with normal volume, e.g. reservoir strain showed a stronger relationship with LGE (R = 0.63 p = 0.001), but not LA volume. Among patients with elevated LA volume, LA LGE became well correlated with LA volumes (R > 0.67, p < 0.003), but not LA strains. This shows that strain indices do reflect fibrosis but more strongly among a group with normal LA volume. Stiffness index was most consistently (i.e. for both elevated and normal volumes) correlated with atrial fibrosis. LGE correlations were mainly stronger than LGE (%) (see Table 2 vs. Table 3), but showed the same trends. For those with observable active function, LGE (mls) was significantly correlated only with active peak strain rate (R = 0.36, p = 0.03) (Table 2), of all active strain parameters.

### Functional correlates to pressure

Of all correlates among those considered (listed in Table 1) pressure was significantly correlated (among the total cohort) only with increased BMI (R = 0.27, p = 0.026), increasing maximum LAVi (R = 0.32, p = 0.0076) and increasing time to peak reservoir strain rate (R = 0.32, p = 0.008) (Fig. 4). No other correlate was found.

**Figure 5:**
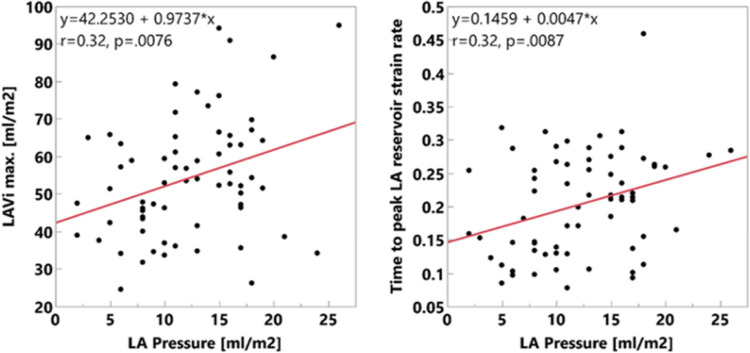
Correlates of mean atrial pressure in the whole cohort (the other correlate, BMI, is not shown).

When patients were categorized by normal vs. elevated LA pressure > 12mmHg and mean values were compared, time to peak reservoir strain was longer in subjects with elevated pressure (0.18 ± 0.07 vs. 0.23 ± 0.07, p = 0.01), conduit time was shorter (0.37 ± 0.06 vs. 0.33 ± 0.06, p = 0.03), and maximum LAVI (ml/m^2^) was larger (59 ± 17 vs. 49 ± 13, p = 0.008) (Fig. 5).

### Functional Correlates with LA volumes

LA reservoir strain was very strongly correlated to many volume indices (LV SVi, LVEF, LVESVi, and all three LA volumes) (Fig. 3), but most strongly with minimum LAVi (R = 0.82, p < 0.001).

## Discussion

There are several new findings from this study. Importantly, this study is the first to identify LA LGE as an index of atrial stiffness, as observed by its moderately strong relationship to “stiffness index” (Pressure over reservoir strain) in all patients (R = 0.59 for LGE), confirmed in subgroups of with normal and elevated volumes. The relationship of LA LGE (a surrogate biomarker of fibrosis) to stiffness index and to strain (especially for patients with normal volumes) is expected based on Eq. 1, and because collagen increases stiffness [5].

While pressure did not correlate to LA strains, there were significant relationships between pressure and maximum LA volumes, and time to peak reservoir strain. The increases in LA volume with pressure of the thin-walled LA is expected; indeed an echocardiographic study found a remarkably similar effect, if slightly weaker (r = 0.24) [9] in 100 patients with hypertrophic cardiomyopathy. The relationship of cardiac timings with pressure is known in other contexts [25]. For example, increasing isovolumic relaxation time (IVRT) [7] has been associated with elevated pressure, as has deceleration timings of early diastolic flow [10] and pulmonary vein flow [15]. In this study, pressure was weakly to moderately associated with timing to peak reservoir strain rate, associated with LA filling speed. An overstretched atrium might be slower to fill,. The finding that strain and pressure are not correlated in the general AF cohort agrees with a recent report comparing atrial strain and pressure [14], where a subset of AF subjects was analyzed separately, with the finding that strain was generally low and the relationship not significant. LA LGE was found to be modestly correlated to pressure in a study of AF subjects [31], which we did not find, possibly because they had a greater range of elevated pressures and did not use mean LAP, as in this study.

Finally, LA strain is strongly correlated to LAEF (R = 0.95) and LA minimum volume (R = 0.82), which questions the uniqueness of the LA reservoir strain metric. A recent study showed that every point on the LA strain vs. time curve is strongly linearly related to LV strain vs. time curve with a slope equal to the ratio of maximal LV and LA volumes [23].

LA LGE volume (mls) showed consistently stronger correlations with strains, stiffness index, and other metrics than LGE (%). The concept of normalizing LGE in mls by volume of atrial myocardium was introduced by Oakes et al.[24] and is analogous to the enhancement percentage used for ventricular LGE. However, in AF where the hallmark of disease is both an enlarged and fibrotic myocardium, LGE (%) may be less useful as a biomarker than LGE (mls), since normalizing elevated LA LGE by an elevated LA volume certainly will obscure abnormal findings.

## Limitations

Although effort was made to exclude patients who were imaged during AF, some patients might have been incorrectly included or excluded in this study. Pressure was measured prior to the ablation procedure, and its reliability is likely to be less than if it were measured for clinical decision-making; furthermore, average pressure was obtained instead of the complete pressure curve and some data may have been acquired during non-sinus rhythm. It is intuitive that pressure changes (i.e. from begin to end LV systole), might be a more relevant correlate of reservoir LA strain magnitude. Additionally, the cohort included fewer patients with very high pressures, compared to other studies [38]. We were not able to use wall-thickness of the LA as another factor in the strain-stiffness-pressure relationship [2]. Increasing the cohort size would have been useful.

## Conclusion

In conclusion, our study of AF patients demonstrates that LA LGE is a marker of stiffness (Pressure/LA reservoir strain). Atrial reservoir strain is linked to LA volume, and thus the relationship of fibrosis to strain is stronger among patients with normal volumes. LA pressure did modestly correlate to maximum LA volume, and with cardiac phasic timing of peak reservoir strain. In contrast to that reported in other cohorts, in AF patients LA strain is not a good correlate of left atrial pressure.

## Figures and Tables

**Figure 6:**
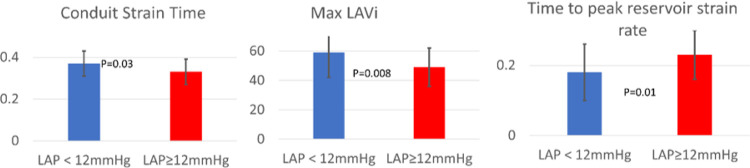
When comparing groups with normal vs. elevated pressure (LAP≥12mmHg, n=33), reservoir and conduit strain times and maximum LAVi were found to be different.

**Table 1:** Patient functional measurements, categorized by LA volumes. The p-value is given for a Student’s t-test between low and high LA volume groups.

	All patients (n = 67)	LAVi min < 30 (n = 32)	LAVi min ≥ 30 (n = 35)	p-value
LV parameters				
LVEDVi [ml/m^2^]	75.8 ± 17.8	75.1 ± 14.3	76.4 ± 20.6	--
LVESVi [ml/m^2^]	35.0 ±14.9	31.1 ± 8.2	38.6 ± 18.5	**0.03**
LVSVi [ml/m^2^]	40.7 ±10.1	43.9 ± 8.1	37.8 ± 10.8	**0.01**
LVEF [%]	54.9 ±11.1	58.9 ± 5.9	51.2 ± 13.3	**0.003**
LVMi [g/m^2^]	54.2 ±14.4	52.8 ± 14.8	55.5 ± 14.1	--
LA Volumes				
LAVi min [ml/m^2^]	33.0 ±16.9	19.0 ± 4.9	45.6 ± 13.5	**< .0001**
LAVi max [ml/m^2^]	54.1 ±15.9	43.0 ± 9.6	64.3 ± 13.5	**< .0001**
LAVi active [ml/m^2^]	41.9 ±14.8	31.1 ± 6.8	51.9 ± 13.0	**< .0001**
LAEF [%]	40.5 ±17.3	55.1 ± 6.9	27.1 ± 12.2	**< .0001**
LAEF active [%]	23.4 ± 7.2	27.4 ± 6.4	19.8 ± 6.0	**< .0001**
LA strains and strain rates				
Reservoir strain [%]	21.0 ± 10.4	29.3 ± 6.3	13.4 ± 7.0	**< .0001**
Conduit strain [%]	14.8 ± 4.4	16.2 ± 4.0	11.8 ± 3.6	**0.001**
Active strain [%]	11.7 ± 4.9	13.3 ± 4.1	8.0 ± 4.5	**0.001**
Reservoir SR [%/s]	0.88 ± 0.38	1.15 ± 0.26	0.62 ± 0.27	**< .0001**
Conduit SR [%/s]	−0.96 ± 0.38	−1.15 ± 0.27	−0.79 ± 0.38	**< .0001**
Active SR [%/s]	−0.89 ± 0.38	−1.04 ± 0.31	−0.58 ± 0.34	**0.0003**
Reservoir time [%]	0.44 ± 0.08	0.42 ± 0.05	0.46 ± 0.10	**0.04**
Conduit length [%]	0.35 ± 0.06	0.35 ± 0.07	0.35 ± 0.06	--
Active time [%]	0.77 ± 0.04	0.77 ± 0.03	0.77 ± 0.05	--
Reservoir SR time [%]	0.20 ± 0.08	0.19 ± 0.07	0.22 ± 0.08	--
Conduit SR time [%]	0.59 ± 0.10	0.55 ± 0.06	0.63 ± 0.12	**0.002**
Active SR time [%]	0.89 ± 0.03	0.89 ± 0.03	0.89 ± 0.04	--
LA remodeling				
Stiffness index [mmHg]	0.90 ± 1.01	0.40 ± 0.20	1.36 ± 1.2	**< .0001**
Mod. Stiffness ind. [mmHgcm]	3.6 ± 4.2	1.4 ± 0.7	5.5 ± 5.1	**< .0001**
Pressure [mmHg]	12.2 ± 5.3	11.3 ± 5.5	13.0 ± 5.0	--
LA LGE [%]	6.2 ± 6.4	5.5 ± 4.8	7.3 ± 8.2	--
LA LGE [ml]	0.73 ± 0.86	0.56 ± 0.58	0.97 ± 1.1	--

**Table 2:** Significant correlates of atrial fibrosis as LA LGE (mls). Spearman rho and p-values (rho (p-value)) are presented. While p-value < 0.05 was used to determine significance, values are presented up to p-value < 0.1. For clarity, LA strain rates are presented as their absolute values. No strain timings or strain rate timings were correlated with LA LGE.

	All patients (n = 41)	LAVi min < 30 (n = 24)	LAVi min ≥ 30 (n = 17)
LV Parameters			
LVEDVi [ml/m^2^]	--	--	--
LVESVi [ml/m^2^]	--	--	--
LVSVi [ml/m^2^]	--	--	--
LVEF [%]	--	--	--
LVMI [g/m^2^]	--	0.38 (0.07)	--
LA volumes			
LAVi min [ml/m^2^]	**0.52 (< 0.001)**	–	**0.67 (0.003)**
LAVi max [ml/m^2^]	**0.41 (0.007)**	–	**0.68 (0.002)**
LAVi active [ml/m^2^]	**0.43 (0.004)**	--	**0.69 (0.01)**
LAEF [%]	**−0.44 (0.004)**	**−0.57 (0.0037)**	--
LAEF active [%]	–	−0.36 (0.08)	--
LA strains and strain rates			
Reservoir strain [%]	**−0.49 (0.001)**	**−0.63 (> 0.001)**	--
Conduit strain [%]	–	**−0.56 (0.004)**	--
Active strain [%]	--	−0.39 (0.06)	--
|Reservoir SR| [%/s]	**−0.43 (0.0050)**	**−0.51 (0.009)**	--
|Conduit SR| [%/s]	**0.38 (0.01)**	**0.59 (0.002)**	--
|Active SR| [%/s]	**−0.36 (0.03)**	**−0.4 (0.048)**	.--
LA remodeling			
Stiffness index [mmHg]	**0.57 (< .0001)**	**0.49 (0.013)**	**0.56 (0.018)**
Pressure [mmHg]	--	--	--

**Table 3:** Significant correlates of atrial fibrosis as relative LA LGE (%). Spearman rho and p-values (rho (p-value)) are presented. While p-value < 0.05 was used to determine significance, values are presented up to p-value < 0.1. For clarity, LA strain rates are presented as their absolute values. No strain or strain rate timings or LV parameter were correlated with LA LGE (%).

	All patients (n = 41)	LAVi min < 30 (n = 24)	LAVi min ≥ 30 (n = 17)
LA volumes			
LAVi min [ml/m^2^]	**0.36 (0.0206)**	--	**0.48 (0.0497)**
LAVi max [ml/m^2^]	**0.32 (0.0440)**	--	**0.61 (0.0100)**
LAVi active [ml/m^2^]	**0.37 (0.0174)**	--	**0.57 (0.0181)**
LAEF [%]	−0.29 (0.0703)	**−0.62 (0.0014)**	--
LAEF active [%]	**−0.33 (0.0378)**	**−0.53 (0.0079)**	--
LA strains and strain rates			
Reservoir strain [%]	**−0.33 (0.0368)**	**−0.55 (0.0049)**	--
Conduit strain [%]	−0.31 (0.0633)	**−0.56 (0.0045)**	--
Active strain [%]	--	--	--
|Reservoir SR| [%/s]	−0.30 (0.0539)	−0.40 (0.0515)	--
|Conduit SR| [%/s]	**−0.36 (0.0200)**	**−0.55 (0.0051)**	--
|Active SR| [%/s]	--	--	--
LA remodeling			
Stiffness index [mmHg]	0.30 (0.0601)	0.	--
Pressure [mmHg]	--	--	--

## Data Availability

The strain analysis package is available upon reasonable request from the authors. The data consists of pressure, strains, functional metrics, and atrial fibrosis, and will be available from the last author upon reasonable request.
